# Editorial: Cardiometabolic diseases and inflammatory responses

**DOI:** 10.3389/fimmu.2024.1384022

**Published:** 2024-03-01

**Authors:** Nadine Suffee, Wilfried Le Goff, Jianmin Chen

**Affiliations:** ^1^ Sorbonne Université, INSERM, Foundation for Innovation in Cardiometabolism and Nutrition (ICAN), UMR_S1166, Paris, France; ^2^ William Harvey Research Institute, Queen Mary University of London, London, United Kingdom; ^3^ Centre for inflammation and Therapeutic Innovation, Queen Mary University of London, London, United Kingdom

**Keywords:** systemic inflammation, heart failure, coronary artery disease, stroke, autoimmune diseases, ascending thoracic aortic aneurysm

Cardiometabolic diseases (CMD) are driven by both metabolic disorders (obesity, insulin resistance, non-alcoholic fatty liver diseases, and atherosclerosis) and chronic inflammation (e.g. diabetes, hypertension and autoimmune diseases), leading to coronary artery disease (CAD), stroke and heart failure (HF) (1–4).

Compelling data from animal models and human intervention studies, such as CANTOS (The Canakinumab Anti-inflammatory Thrombosis Outcome Study) (5), indicate that addressing lingering systemic inflammation in humans could potentially yield benefits in mitigating CMD. While the precise mechanisms connecting chronic inflammation and CMD remain a subject of active research, recognised pathways in chronic inflammatory conditions encompass immune activation, systemic inflammation, lipoprotein abnormalities, and insulin resistance. These factors contribute to the susceptibility of individuals to CMD (2).

Today, innovative approaches and the view of a systemic dimension of the physiology can deeply decipher mechanisms. The aim of our topic is to highlight a seminal insight into the impact of immune response, metabolic regulation and the development of cardiovascular physiopathology. Our objectives are to: 1) introduce new concepts in cardiovascular research, highlighting novels tools for precise analysis; 2) demonstrate that CMD are not limited to a local perspective but they encompass a systemic dimension that impacts physiology and function of the organs and tissue.

Single-cell RNA sequencing (scRNA-Seq) tool has enabled elaboration of an atlas of immune cells involved in the formation of the atherosclerosis plaques. Ge et al. have deciphered major groups of immune cells from lymphoid and myeloid linages. Their results have shown a dynamic relationship between immune cells and tissue remodeling. This tool exemplifies the capacity to study a broad spectrum of immune cell clusters. Indeed, their study illustrated that inflammatory macrophages affect stability of plaques whereas B-cells contribute to plaque stability.

ScRNA-Seq analysis was also employed by Tian et al. to study the role of immunogenic cell death in ascending thoracic aortic aneurysms (ATAA), which was poorly understood. Analysis of 10 cell types (monocytes, macrophages, CD4^+^ T and natural killer cells, mast cells, B/plasma B cells, fibroblasts, endothelial cells, cytotoxic CD8^+^ T cells, vascular smooth muscle cells and mature dendritic cells) from 11 individuals (8 ATAA and 3 controls) revealed the importance of endothelial cells in immunogenic cell death as well as their potential role in T-cell infiltration and myeloid cell infiltration in ATAA. In addition, this study proposes ACKR1 and CXCL12 as potential new therapeutic targets in ATAA.

Epicardial adipose tissue (EAT) is recognised as a pivotal immune organ in HF pathogenesis. However, immune cell characteristics within HF patient EAT have been understudied. Zhang et al. used public scRNA-Seq data and found EAT enriched in immune activation-related genes and T lymphocytes compared to subcutaneous adipose tissue. HF patient EAT showed heightened T lymphocyte activation. The study also unveiled clonally expanded IFN-γ^+^ effector memory T lymphocytes in HF patient EAT. These findings provide valuable insights into the immune environment in EAT and its potential implications for heart failure.

Another novel tool is also emerging in cardiomyopathies detection. Imagery is a promising modality, particularly in identifying high intensity signal *in situ* that could provide new insight for myocardium rescue. In addition, the capability to translate research data to clinical application holds significant potential. Wu et al have shown the cardiac magnetic resonance imaging (MRI) could aid in the detection of inflammation in the remote myocardium in myocardial infarction (MI) porcine models and small animal model. They correlated their imagery data with inflammation detection. Their data have shown that through imaging, it is possible to detect abnormal signals in both of the early stages and over time.

In the last decade, the systemic dimension has emerged as a predictor of cardiovascular diseases (CVD), alongside cardiomyopathies. Resident immunomodulation, metabolism and mitochondria of the cardiac tissue are highly responsive to the environment. Experiments involving factors such as gut microbial, the circadian clock or hypoxia have been shown to impact tissue function, leading to conditions such as arrhythmias, along with tissue remodeling notably with occurrence of fibrosis. These factors are associated to cardiometabolic disorders or myocarditis (Adzika et al.), (Jin et al.), (Gao et al.). Similarly, systemic inflammation, immune cells and oxidative stress also contribute to the pathogenesis of hypertension, which is a significant risk factor for CVD, through vascular inflammation and microvascular remodelling (Zhang et al.).

Indeed, strong evidence from epidemiological studies suggests associations between CVD and systemic cytokines, though causality remains uncertain. To investigate this, Wei et al. conducted a Mendelian randomisation study using summary statistics from genome-wide association studies (GWAS) of cytokines and CVD, revealing causal effects of four cytokines (IL-1ra, MCSF, SeSelectin, SCF) on CAD and associations between two cytokines (IL-2ra, IP-10) with heart failure, and two cytokines (MCP-3, SeSelectin) with atrial fibrillation. These findings offer insights for novel therapeutic strategies targeting specific cytokines to prevent and treat certain CVDs.

Similarly, circulating white blood cells (WBCs) were largely demonstrated to be associated with CMD including metabolic syndrome (MetS), however the role of insulin resistance in this association is unknown. A cross-sectional study in 7,014 patients from the China Health and Nutrition Survey (CHNS) conducted by Ren et al. showed that normal WBC count levels protect against MetS independently of the presence of insulin resistance and proposed a performant multilayer perceptron algorithm to predict MetS. Then WBC count may be used as a potential risk marker in the identification and the prevention of MetS.

In recent years, the systemic immune-inflammation index (SII) has gained attention for its potential in predicting clinical outcomes, particularly in cancer patients. However, its applicability to stroke patients was unclear. To address this, Huang et al. conducted a systematic review and meta-analysis encompassing 19 retrospective studies involving 18,609 stroke patients. It demonstrated that a higher SII was significantly associated with poor outcomes, mortality, and the incidence of haemorrhagic transformation. Therefore, SII may offer a predictive tool for stroke patients.

Patients with inflammatory arthritis, including gout, psoriasis, and rheumatoid arthritis, face an elevated risk of CMD, independent of traditional cardiovascular risk factors. This connection is likely attributed to local and systemic inflammatory responses (2, 6, 7). Consequently, EULAR recommends cardiovascular risk screening for all patients with inflammatory arthritis and emphasises the role of rheumatologists in risk evaluation in 2022 (8). Wang et al. demonstrated that subclinical atherosclerosis, specifically tophi and carotid plaque, independently predicted MACE in gout patients, even after adjusting for conventional cardiovascular risk factors. They also showed that the frequency of gout flares in the previous year of the baseline was linked with subsequent MACE. Gout flares feature acute inflammation with NLRP-3 inflammasome activation, potentially linked to plaque instability and cardiovascular events through inflammatory cell activation and oxidative stress (9, 10), possibly explaining cardiovascular events after gout flares.

In the context of atherosclerosis, several studies reported the interactions between NLRP3 and PCSK9, a protein involved in the degradation of the low-density lipoprotein receptor in hepatocytes, which may contribute to the inflammatory response occurring during atherogenesis. Interestingly, both NLRP3 and PCKS9 are therapeutic targets in atherosclerotic CVD. In a very interesting review article, Wang et al. describe the mechanisms underlying PCSK9 and NLRP3 inflammasome activation and propose new therapeutic insights in CVD.

Finally, patients with HIV treated with a long-term antiretroviral therapy (ART) displayed a higher incidence and prevalence of CMD in which immune responses to co-infections may contribute. The study of circulating CX3CR1^+^, GPR56^+^, and CD57^+/-^ T cells (termed CGC^+^), a surface marker combination suggestive of antiviral activity, in a cohort of 134 patients with HIV co-infected with cytomegalovirus (CMV) indicated that CGC^+^ CD4^+^ T cells were associated with diabetes, coronary artery calcification and non-alcoholic fatty liver disease, suggesting that anti-CMV therapies might reduce CMD in such patients (Wanjalla et al.).

In summary, the collection of the articles in this Research Topic delves into the intricate relationship between CMD, chronic inflammation, and immune responses, elucidating their roles in conditions such as CAD, stroke, and HF. It highlights the significance of addressing systemic inflammation which may provide potential benefits in mitigating CMD. Cutting-edge tools like scRNA-Seq offer insights into immune cell dynamics in diseases like atherosclerosis and ATAA. Furthermore, innovative approaches such as cardiac MRI show promise in detecting inflammation in MI. Additionally, the review underscores the systemic dimension of CMD, emphasising interactions between immunomodulation, metabolism, and cardiac tissue responsiveness. Studies exploring cytokine associations with CMD and the predictive value of WBC counts further enrich our understanding. This interdisciplinary approach opens avenues for novel therapeutic strategies and underscores the importance of considering systemic immune responses in CMD management.

## Author contributions

NS: Writing – original draft, Writing – review & editing. WLG: Writing – original draft, Writing – review & editing. JC: Writing – original draft, Writing – review & editing.

## References

[1] Mechanick JI Farkouh ME Newman JD Garvey WT. Cardiometabolic-based chronic disease, addressing knowledge and clinical practice gaps: JACC state-of-the-art review. J Am Coll Cardiol. (2020) 75:539–55. doi: 10.1016/j.jacc.2019.11.046. PMC8168371 32029137

[2] Aksentijevich M Lateef SS Anzenberg P Dey AK Mehta NN. Chronic inflammation, cardiometabolic diseases and effects of treatment: Psoriasis as a human model. Trends Cardiovasc Med. (2020) 30:472–8. doi: 10.1016/j.tcm.2019.11.001. PMC7428846 31837960

[3] Urbain F Ponnaiah M Ichou F Lhomme M Materne C Galier S. Impaired metabolism predicts coronary artery calcification in women with systemic lupus erythematosus. EBioMedicine. (2023) 96:104802. doi: 10.1016/j.ebiom.2023.104802. 37725854 PMC10518349

[4] Cho S Ying F Sweeney G. Sterile inflammation and the NLRP3 inflammasome in cardiometabolic disease. BioMed J. (2023) 46:100624. doi: 10.1016/j.bj.2023.100624. 37336361 PMC10539878

[5] Everett BM Cornel JH Lainscak M Anker SD Abbate A Thuren T. Anti-inflammatory therapy with canakinumab for the prevention of hospitalization for heart failure. Circulation. (2019) 139:1289–99. doi: 10.1161/CIRCULATIONAHA.118.038010. 30586730

[6] Choi HK Curhan G. Independent impact of gout on mortality and risk for coronary heart disease. Circulation. (2007) 116:894–900. doi: 10.1161/CIRCULATIONAHA.107.703389. 17698728

[7] Chen J Norling LV Cooper D. Cardiac dysfunction in rheumatoid arthritis: The role of inflammation. Cells. (2021) 10. doi: 10.3390/cells10040881. PMC8070480 33924323

[8] Smolen JS Landewé RBM Bergstra SA Kerschbaumer A Sepriano A Aletaha D. EULAR recommendations for the management of rheumatoid arthritis with synthetic and biological disease-modifying antirheumatic drugs: 2022 update. Ann Rheum Dis. (2023) 82:3–18. doi: 10.1136/ard-2022-223356corr1. 36357155

[9] Martinon F Pétrilli V Mayor A Tardivel A Tschopp J. Gout-associated uric acid crystals activate the NALP3 inflammasome. Nature. (2006) 440:237–41. doi: 10.1038/nature04516. 16407889

[10] Ionita MG van den Borne P Catanzariti LM Moll FL de Vries J-PPM Pasterkamp G. High neutrophil numbers in human carotid atherosclerotic plaques are associated with characteristics of rupture-prone lesions. Arterioscler Thromb Vasc Biol. (2010) 30:1842–8. doi: 10.1161/ATVBAHA.110.209296. 20595650

